# Corticosteroid therapy for coronavirus disease 2019-related acute respiratory distress syndrome: a cohort study with propensity score analysis

**DOI:** 10.1186/s13054-020-03340-4

**Published:** 2020-11-10

**Authors:** Chaomin Wu, Dongni Hou, Chunling Du, Yanping Cai, Junhua Zheng, Jie Xu, Xiaoyan Chen, Cuicui Chen, Xianglin Hu, Yuye Zhang, Juan Song, Lu Wang, Yen-cheng Chao, Yun Feng, Weining Xiong, Dechang Chen, Ming Zhong, Jie Hu, Jinjun Jiang, Chunxue Bai, Xin Zhou, Jinfu Xu, Yuanlin Song, Fengyun Gong

**Affiliations:** 1 Department of Pulmonary and Critical Care Medicine, QingPu Branch of Zhongshan Hospital Affiliated to Fudan University, Shanghai, China; 2 Department of Pulmonary and Critical Care Medicine, Zhongshan Hospital, Fudan University, Shanghai, China; 3 Infection Division, Wuhan Jin Yin-Tan Hospital, Wuhan, China; 4 Department of Urology, Shanghai General Hospital, Shanghai Jiao Tong University School of Medicine, Shanghai, China; 5 Department of Infectious Diseases, Fengxian Guhua Hospital, Shanghai, China; 6 Department of Gastroenterology, Shanghai First People’s Hospital, Shanghai Jiao Tong University School of Medicine, Shanghai, China; 7 Department of Respiratory and Critical Care Medicine, Shanghai Ninth People’s Hospital, Shanghai Jiao Tong University School of Medicine, Shanghai, China; 8 Department of Critical Care Medicine, Ruijin Hospital, Shanghai Jiao Tong University School of Medicine, Shanghai, China; 9 Department of Critical Care Medicine, Zhongshan Hospital, Fudan University, Shanghai, China; 10 Department of Pulmonary Medicine, Shanghai First People’s Hospital, Shanghai Jiao Tong University School of Medicine, Shanghai, China; 11 Department of Respiratory and Critical Care Medicine, Shanghai Pulmonary Hospital, Shanghai, China; 12 Shanghai Respiratory Research Institute, Shanghai, China; 13 National Clinical Research Center for Aging and Medicine, Huashan Hospital, Fudan University, Shanghai, China; 14 Jinshan Hospital of Fudan University, Shanghai, China

**Keywords:** Corticosteroids, Coronavirus disease 2019, Severe acute respiratory syndrome coronavirus 2, Mortality, Propensity score, Methylprednisolone

## Abstract

**Background:**

The impact of corticosteroid therapy on outcomes of patients with coronavirus disease 2019 (COVID-19) is highly controversial. We aimed to compare the risk of death between COVID-19-related ARDS patients with corticosteroid treatment and those without.

**Methods:**

In this single-center retrospective observational study, patients with ARDS caused by COVID-19 between January 20, 2020, and February 24, 2020, were enrolled. The primary outcome was 60-day in-hospital death. The exposure was prescribed systemic corticosteroids or not. Time-dependent Cox regression models were used to calculate hazard ratios (HRs) and 95% confidence intervals (CIs) for 60-day in-hospital mortality.

**Results:**

A total of 382 patients [60.7 ± 14.1 years old (mean ± SD), 61.3% males] were analyzed. The median of sequential organ failure assessment (SOFA) score was 2.0 (IQR 2.0–3.0). Of these cases, 94 (24.6%) patients had invasive mechanical ventilation. The number of patients received systemic corticosteroids was 226 (59.2%), and 156 (40.8%) received standard treatment. The maximum dose of corticosteroids was 80.0 (IQR 40.0–80.0) mg equivalent methylprednisolone per day, and duration of corticosteroid treatment was 7.0 (4.0–12.0) days in total. In Cox regression analysis using corticosteroid treatment as a time-varying variable, corticosteroid treatment was associated with a significant reduction in risk of in-hospital death within 60 days after adjusting for age, sex, SOFA score at hospital admission, propensity score of corticosteroid treatment, comorbidities, antiviral treatment, and respiratory supports (HR 0.42; 95% CI 0.21, 0.85; *p* = 0.0160). Corticosteroids were not associated with delayed viral RNA clearance in our cohort.

**Conclusion:**

In this clinical practice setting, low-dose corticosteroid treatment was associated with reduced risk of in-hospital death within 60 days in COVID-19 patients who developed ARDS.

## Background

The World Health Organization (WHO) declared that the outbreak of severe acute respiratory syndrome coronavirus 2 (SARS-CoV-2) constitutes a pandemic [1]. Since the first confirmed case on January 22, 2020, the virus has been emerged in 216 countries. More than 14,765,000 laboratory-confirmed cases were reported, with an average mortality approaching 4.1% as of July 22, 2020 [2]. The spread of SARS-CoV-2 has led to serious socioeconomic consequences worldwide.

Currently, there is no specific treatment or vaccine for coronavirus disease 2019 (COVID-19). Up to 29% of the COVID-19 patients developed acute respiratory distress syndrome (ARDS) [3–5] as a consequence of cytokine storm. ARDS was the major cause of morbidity [6]. Adjunctive corticosteroids may be theoretically beneficial [7], and has been widely used by clinicians to suppression of hyperinflammation in COVID-19 patients, especially those with critical illness [3, 5, 8]. However, there was comprehensive controversy on its efficacy [9, 10], due to the results of observational studies that showed corticosteroid treatment was associated with increased mortality and nosocomial infections for influenza and delayed virus clearance for severe acute respiratory syndrome (SARS), Middle East respiratory syndrome (MERS) [11].

In the early pandemic of COVID-19, lower mortality was reported among the critically ill subgroup of SARS patients treated with corticosteroids in a retrospective study [12]. Since then, evidence is growing that corticosteroid treatment is beneficial for some COVID-19 patients. The Randomized Evaluation of COVID-19 Therapy (RECOVERY) trial from the UK reported reduced mortality in patients treated with oral or intravenous dexamethasone 6 mg/d for up to 10 days [13]. The efficacy was significant only in severe patients who receiving oxygen or invasive mechanical ventilation. A prospective meta-analysis of 7 RCTs of critically ill patients with COVID-19 also showed the association between systemic corticosteroids and lower all-cause mortality [14]. Other two randomized controlled trials (RCTs) did not show benefit on mortality from intravenous dexamethasone or methylprednisolone treatment, but corticosteroid treatment increased ventilator-free days and number of days alive in patients with moderate to severe ARDS [15, 16]. A meta-analysis of corticosteroids on the mortality of ARDS showed beneficial effects of corticosteroids on short-term mortality may be counteracted by the delayed onset of adverse effects [17], such as secondary infection due to immunosuppression and altered tissue repair. A follow-up period of 28 days in these randomized trials may underestimate the late adverse effects of corticosteroids on all-cause mortality. In addition, most of the aforementioned trials studied dexamethasone, and evidence for methylprednisolone was limited.

In this observational study, we thoroughly examined the associations of corticosteroid treatment with 60-day in-hospital mortality among a population of COVID-19 patients who have developed ARDS.

## Materials and methods

### Study design and patients

This single-center, retrospective, cohort study was conducted at the Jin Yin-tan Hospital, Wuhan, China. We identified all adult patients with confirmed COVID-19 according to WHO interim guidance [18] and patients were admitted between January 20, 2020, and February 24, 2020. Then, we identified those who developed ARDS according to the WHO definition for analysis [18]. Among these patients, 84 have been described previously by Wu et al. [8], and 91 participated in the open-label trial of Lopinavir–Ritonavir [19]. To avoid the influence of early mortality before cortical steroid presenting treatment efficacy, patients who died or discharged within 2 days on hospital admission were excluded. Other exclusion criteria were: (1) participating in any double-blind clinical trial, (2) under long-term corticosteroid therapy for at least 1 month as part of treatment for chronic underline diseases, or (3) could not provide valid medical history because of mental disease. The Jin Yin-tan Hospital Ethics Committee approved the study (No. KY-2020-44.01) and granted a waiver of informed consent from study participants.

In Jin Yin-tan hospital, systemic corticosteroids were considered if patients had progressive respiratory failure or laboratory findings indicated the presence of hyperinflammatory response. In patients receiving mechanical ventilation, preventive ventilation strategy of tidal volume 4–8 mL/kg of predicted body weight, inhale positive airway pressure < 30 cmH_2_O, and PEEP > 5 cmH_2_O was followed.

### Data collection

Administration of corticosteroids was defined as systemic use (oral or intravenous) of corticosteroids, including methylprednisolone, dexamethasone, hydrocortisone, and prednisone. The primary outcome was 60-day in-hospital mortality. Patients were followed to death or discharge from hospital up to 60 days since hospital admission (last clinical outcome was observed on March 21, 2020). The secondary outcomes were time to SARS-CoV-2 viral clearance since symptom onset. Data on demographics, medical history, laboratory findings, chest radiology, medication use, and clinical outcomes were extracted retrospectively from electronic medical records using a standardized data collection form. All data were checked independently by two physicians (DH and XC). From January 11, 2020, to monitor the clearance of viral RNA, SARS-CoV-2 RNA was tested using polymerase chain reaction from throat-swab specimens for every other day after clinical remission of symptoms, including fever, cough, and dyspnea [8]. Viral clearance was defined as two consecutive negative results. The definitions of ARDS and other diseases were described in eMethods (Additional file 1).

### Statistical analysis

Baseline characteristics were compared between patients with and without corticosteroid treatment. Data were reported as percentage for categorical variables and as mean ± standard deviation (SD) or median with interquartile range (IQR, 25–75%) for continuous variables. Categorical variables were compared by Fisher’s exact test or Pearson chi-square test, as appropriate, and continuous variables were compared by Mann–Whitney *U* test or Student’s *t* test.

### Propensity score adjustment

To reduce the effect of steroids treatment bias and potential confounding factors, we performed propensity score analysis [20] to adjust the differences in baseline characteristics. For each patient, a propensity score indicating the likelihood of receiving systemic corticosteroid treatment was calculated by a logistic regression model. The model included 10 pre-selected baseline variables based on the clinical guidelines from National Health Commission of China, which recommended corticosteroids for patients with progressive respiratory failure and hyperinflammation response. Specifically, SpO_2_/FiO_2_ ratio and respiratory rate were included for indicating severe respiratory failure; temperature, heart rate, SOFA score, blood lymphocyte count, blood neutrophil count, and level of CRP at hospital admission were included for indicating systemic inflammatory response syndrome; age and sex were included as basic characteristics of each patient. The outcome variable was whether or not the patient received corticosteroid therapy in the current hospital stay. Goodness of fit was evaluated by the c-statistic and the Hosmer–Lemeshow test.

### Cox proportional-hazard regression model

The effect of corticosteroid treatment on risk of 60-day in-hospital all-cause death was analyzed using a series of Cox proportional-hazard regression models. First, we constructed a univariable Cox regression model on hospital death by 60 days since hospital admission with corticosteroid treatment treated as a time-varying covariate. Then, we constructed a multivariable Cox model of 60-day hospital death with corticosteroid treatment as time-varying covariate and incorporated the individual propensity score into the model as a covariable to calculate the propensity adjusted hazard ratio (HR). In the final model, the effects of corticosteroids on 60-day in-hospital death were adjusted for propensity score of corticosteroid treatment, as well as the following pre-selected covariates: age, sex, sequential organ failure assessment (SOFA) score at hospital admission, comorbidities (diabetes, hypertension, coronary artery disease, chronic pulmonary disease, chronic renal or liver disease, solid malignant tumor, hematologic malignancy, and immunosuppressive status), antiviral treatment (Lopinavir–Ritonavir, oseltamivir, and ganciclovir), and respiratory supports (high-flow oscillation oxygen, noninvasive mechanical ventilation, and invasive mechanical ventilation) in hospital [9].

### Sensitivity analysis

Several sensitivity analyses were performed to assess the robustness of our findings. To test whether the findings were influenced by the time point of baseline variables used in propensity score analysis, Cox proportional-hazard regression models were repeated in sensitivity analyses: (1) comparing patients receiving corticosteroids within 2 days after hospital admission versus no corticosteroids; (2) comparing patients receiving corticosteroids versus no corticosteroids using ARDS onset date as baseline, where values of SpO_2_/FiO_2_ ratio, respiratory rate, temperature, heart rate, respiratory rate, SOFA score, blood lymphocyte count, blood neutrophil count, and level of CRP at ARDS onset were used; (3) comparing patients receiving corticosteroids within 2 days after ARDS diagnosis versus no corticosteroids with ARDS onset as baseline. To test whether the findings might be influenced by ARDS definition, we conducted survival analysis using the same model among patients diagnosed with ARDS by Berlin definition [21]. The difference between Berlin Definition and WHO definition was that the latter included patients with SpO_2_/FiO_2_ ≤ 315 when PaO_2_ is not available, and did not restrict to ventilated patients.

### Viral shedding and inflammation

Differences in the time to SARS-CoV-2 RNA clearance were analyzed using Cox proportional-hazard regression adjusted for the same covariables with corticosteroid therapy as a time-varying covariate. Patients died without viral shedding or discharged alive before they had two consecutive negative SARS-CoV-2 RNA tests were censored.

The associations between c-reactive protein with corticosteroids treatment after ARDS onset were analyzed using the interaction between corticosteroids and days after ARDS onset based on a linear regression model.

Results were analyzed with SAS (version 9.4, SAS Institute, Cary, NC). Unadjusted and adjusted hazard ratios and their 95% confidence intervals (Cis) were reported. Two-sided P values less than 0.05 were considered statistically significant.

## Results

A total of 1147 patients with COVID-19 were screened for the study. Forty patients were excluded for participating in any double-blind clinical trial (*n* = 15), death or discharge from the hospital within 2 days after hospital admission (*n* = 21), underwent long-term corticosteroid therapy for chronic kidney disease or rheumatic disease (*n* = 3), or no valid medical history provided because of mental disease (*n* = 1). From 1107 patients remained, 382 patients were identified as ARDS (Additional file 1: Fig. S1).

Baseline characteristics of the ARDS patients at hospital admission by receiving systemic corticosteroid treatment are shown in Table 1. In the entire cohort, the mean age was 60.7 ± 14.1 years, and 234 (61.3%) patients were male. 147 (38.5%) were treated with NIMV, 94 (24.6%) with IMV, and 11 (2.9%) with ECMO. All but one of the patients reached end point of decease (53.1%) or discharge (46.6%) during the follow-up period of 60 days. The median duration of follow-up was 12.0 (IQR 7.0–18.0) days. A total of 226 (59.2%) ARDS patients had a prescription of systemic corticosteroids. Corticosteroids were more likely prescribed to the younger (*p* = 0.0077) and males (*p* = 0.0135). Corticosteroids group had lower lymphocyte count and higher levels of CRP and lactate dehydrogenase at hospital admission than non-corticosteroids group, indicating a propensity in prescribing corticosteroids to patients with more severe immune dysfunction and inflammatory response (Table 1). The 60-day hospital death in patients who ever used corticosteroids was higher than the patients who did not use corticosteroids [135 (59.7%) vs. 68 (43.6%), *p* = 0.0019]. However, the median survival duration was longer in corticosteroid group [19.0 (IQR 15.0–21.0) vs. 15.0 (IQR 12.0–23.0), *p* = 0.0239].

**Table 1:** Characteristics of patients with acute respiratory distress syndrome associated with coronavirus disease 2019

Characteristics	All	Corticosteroids	No corticosteroids	*p *value
*N*	382 (100.0)	226 (59.2)	156 (40.8)	–
Age (year)	60.7 ± 14.1	59.1 ± 14.0	63.0 ± 14.0	0.0077
Male sex	234 (61.3)	150 (66.4)	84 (53.8)	0.0135
Smoking history	35 (9.2)	24 (10.6)	11 (7.1)	0.2347
Days from onset at hospital admission	11.0 (8.0–15.0)	10.0 (7.0–14.0)	12.0 (9.0–16.5)	0.0029
Medical history				
Chronic pulmonary disease	20 (5.2)	12 (5.3)	8 (5.1)	0.9376
Hypertension	136 (35.6)	79 (35.0)	57 (36.5)	0.7508
Diabetes	67 (17.5)	36 (15.9)	31 (19.9)	0.3193
Chronic liver disease	15 (3.9)	11 (4.9)	4 (2.6)	0.2546
Chronic renal disease	6 (1.6)	2 (0.9)	4 (2.6)	0.1945
Cardiovascular disease	28 (7.3)	12 (5.3)	16 (10.3)	0.0682
Malignant tumor	12 (3.1)	7 (3.1)	5 (3.2)	0.9527
Hematological malignant tumor	2 (0.6)	1 (0.5)	1 (0.7)	0.8339
Immunosuppressive conditions	14 (3.7)	9 (4.0)	5 (3.2)	0.6911
SOFA score at hospital admission	2.0 (2.0–3.0)	2.0 (2.0–3.0)	2.0 (2.0–3.0)	0.1383
Corticosteroid therapy before hospital admission	40 (10.5)	28 (12.4)	12 (7.7)	0.1405
Vital signs at hospital admission				
Temperature (°C)	36.8 ± 0.7	36.9 ± 0.8	36.7 ± 0.5	0.0069
Heart rate (min^−1^)	90.9 ± 15.3	92.9 ± 16.3	88.0 ± 13.1	0.0018
Respiratory rate (min^−1^)	24.0 ± 6.3	24.5 ± 7.1	23.3 ± 5.0	0.0609
Laboratory findings				
Blood leukocyte count (× 10^9^/L)	8.1 (5.2–11.3)	8.4 (5.1–11.7)	7.5 (5.4–10.0)	0.2593
Lymphocyte count (× 10^9^/L)	0.7 (0.5–1.0)	0.6 (0.5–0.8)	0.8 (0.6–1.1)	< 0.0001
Neutrophil count (× 10^9^/L)	6.9 (4.0–10.2)	7.4 (4.2–10.7)	5.7 (4.0–8.7)	0.0508
SpO_2_/FiO_2_	229.3 (175.5–352.4)	218.6 (170.0–366.7)	241.5 (184.0–332.8)	0.2133
CRP (mg/L)	89.0 (38.0–159.9)	96.7 (45.9–160.0)	68.7 (28.6–138.5)	0.0026
D-dimer (mg/L)	1.5 (0.7–8.0)	1.5 (0.6–9.5)	1.5 (0.7–7.1)	0.9913
Lactate dehydrogenase (U/L)	409.0 (304.0–545.0)	429.0 (320.0–569.0)	386.5 (277.0–509.5)	0.0124
Bilateral involvement	358 (93.7)	214 (94.7)	144 (92.3)	0.3455
Severity of ARDS^a^				
Mild	120 (31.4)	60 (26.5)	60 (38.5)	0.0297
Moderate	157 (41.1)	99 (43.8)	58 (37.2)	
Severe	105 (27.5)	67 (29.6)	38 (24.4)	
Antivirus drugs				
Lopinavir	91 (24.0)	72 (31.9)	19 (12.4)	< 0.0001
Ganciclovir	32 (8.4)	19 (8.4)	13 (8.5)	0.9754
Interferon	103 (27.2)	65 (28.8)	38 (24.8)	0.3994
Oseltamivir	64 (16.9)	52 (23.0)	12 (7.8)	0.0001
Antibiotics	371 (97.1)	224 (99.1)	147 (94.2)	0.0126
Respiratory support during hospital stay				
High-frequency oscillation ventilation	146 (38.8)	100 (45.2)	46 (29.7)	0.0023
NIMV	147 (38.5)	104 (46.0)	43 (27.6)	0.0003
IMV	94 (24.6)	59 (26.1)	35 (22.4)	0.4130
ECMO	11 (2.9)	8 (3.5)	3 (1.9)	0.3530
Hyperglycemia	32 (8.4)	20 (8.8)	12 (7.7)	0.6882
In-hospital 60-day mortality	203 (53.1)	135 (59.7)	68 (43.6)	0.0019
In-hospital days for all patients	12.0 (7.0–18.0)	14.0 (9.0–21.0)	10.0 (6.0–13.0)	< 0.0001
In-hospital days for survivors	13.0 (10.0–19.0)	16.0 (11.0–24.0)	11.0 (8.5–15.0)	< 0.0001
Median survival time (days)	18.0 (15.0–20.0)	19.0 (15.0–21.0)	15.0 (12.0–23.0)	0.0239
Duration of viral shedding from symptom onset (days)	18.0 (14.0–23.0)	19.0 (14.0–23.0)	18.0 (14.0–24.0)	0.7217

Data are *n* (%), mean (SD), or median (IQR). For continuous variables, *t *test or Mann–Whitney *U* test was used to calculate the *p* value unless otherwise noted. For categorical variables, chi-square test was used to calculate the *p* value unless otherwise noted

*ARDS* acute respiratory distress syndrome, *SOFA* sequential organ failure assessment, *CRP* c-reactive protein, *MV* mechanical ventilation, *NIMV* noninvasive mechanical ventilation, *IMV* invasive mechanical ventilation, *ECMO* extracorporeal membrane oxygenation, *SpO*_*2*_ pulse oxygen saturation, *FIO*_*2*_ fraction of inspired oxygen. ARDS was defined according to World Health Organization interim guidance

^a^PaO_2_/FiO_2_ was estimated from SpO_2_/FiO_2_ if PaO_2_/FiO_2_ was not available [37]

Among patients prescribed corticosteroids, methylprednisolone was the most frequently administered corticosteroids (213/226, 94.2%) (Table 2). Corticosteroid treatment lasted for 7.0 (IQR 4.0–12.0) days in total. The maximum dose in methylprednisolone equivalent was 80.0 (IQR 40.0–80.0) mg per day and duration of maximum dose was 3.0 (IQR 2.0–5.0) days. Corticosteroids were initiated 13.0 (IQR 10.0–16.0) days after symptom onset. 82.3% (186/226) of the patients received corticosteroids started the therapy within 2 days after ARDS diagnosis. Survivors had shorter duration from symptom onset to corticosteroids [11.0 (9.0–14.0) vs. 14.00 (IQR 11.0–18.0), *p* = 0.0031] and had earlier initiation of corticosteroids with regard to the date of ARDS onset [0.0 (IQR − 1.0 to 1.0) vs. 1.00 (IQR 0.0–2.0), *p* = 0.0102] when compared with non-survivors. Clinical characteristics of survivors and non-survivors received corticosteroid are summarized in eTable 1 (Additional file 1).

**Table 2:** Administration of corticosteroids, stratified by outcome

	All (*n* = 226)	Non-survivors (*n* = 135)	Survivors (*n* = 91)	*p *value
Corticosteroid prescribed				
Methylprednisolone	213 (94.2)	132 (62.0)	83 (38.0)	0.0004
Prednisolone	41 (18.1)	11 (73.2)	30 (26.8)	0.0007
Dexamethasone	5 (2.2)	4 (80.0)	1 (20.0)	0.4470
Maximum dose (methylprednisolone equivalent, mg)	80.0 (40.0–80.0)	80.0 (40.0–80.0)	80.0 (40.0–80.0)	0.0821
Days of corticosteroid treatment	7.0 (4.0–12.0)	6.0 (3.0–11.0)	9.0 (5.0–12.0)	0.0069
Days of maximum dose	3.0 (2.0–5.0)	3.0 (1.0–5.0)	4.0 (2.0–5.0)	0.0287
Days from symptom onset to corticosteroid treatment	13.0 (10.0–16.0)	14.0 (11.0–18.0)	11.0 (9.0–14.0)	0.0031
Days from hospital admission to corticosteroid treatment	1.0 (0.0–3.0)	1.0 (0.0–4.0)	1.0 (0.0–2.0)	0.1892
Days from ARDS to corticosteroid treatment	0.0 (0.0–2.0)	1.0 (0.0–2.0)	0.0 (− 1.0 to 1.0)	0.0102
Days from ventilation to corticosteroid treatment	− 1.0 (− 3.0 to 0.0)	− 1.0 (− 3.0 to 0.0)	− 2.0 (− 4.0 to 1.0)	0.7576

Data are n (%) or medium (IQR). For continuous variables, *t *test or Mann–Whitney *U* test was used to calculate the *p* value unless otherwise noted. For categorical variables, chi-square test was used to calculate the *p* value unless otherwise noted

In the logistic regression model generating propensity score, the pre-selected variables most closely correlated with prescription of systemic corticosteroids included age, blood lymphocyte count, heart rate and CRP (eTable 2, Additional file 1). The multivariable regression model of propensity for corticosteroid treatment had area under the receiver operating characteristic curve (ROC) of 0.71.

**Fig. 1:**
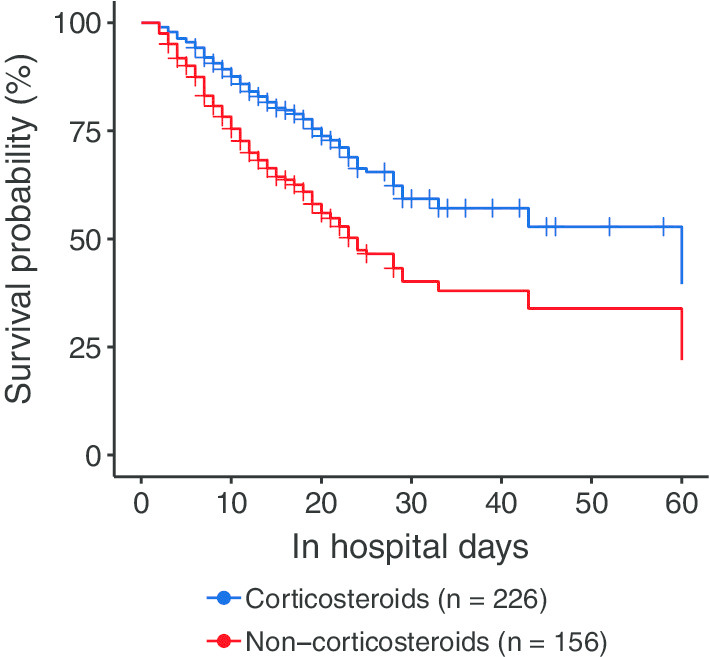
Estimated survival probability of multivariable Cox regression model with time-dependent corticosteroid treatment. Cox regression model with corticosteroid treatment was time-varying variable, adjusting for age, sex, SOFA score at hospital admission, propensity score of corticosteroid treatment, comorbidities (diabetes, hypertension, chronic pulmonary disease, chronic renal or liver disease, solid malignant tumor, hematologic malignancy, and immunosuppressive status), antiviral treatment (Lopinavir–Ritonavir, oseltamivir, and ganciclovir), and respiratory supports (high-flow oscillation oxygen, noninvasive mechanical ventilation, and invasive mechanical ventilation). Propensity score was calculated by a non-parsimonious logistic regression model that included: age, sex, SOFA score, temperature, respiratory rate, SpO_2_/FiO_2_ ratio, blood lymphocyte count, blood neutrophil count, and level of c-reactive protein at hospital admission. ARDS, acute respiratory distress syndrome; SOFA, sequential organ failure assessment

In survival analysis, univariable time-dependent Cox regression model showed the prescription of corticosteroids was associated with a lower risk of death (HR 0.48; 95% CI 0.25, 0.93; *p* = 0.0285) (Table 3). In full model adjusted for age, sex, SOFA score, propensity score, comorbidities, antiviral drugs, and respiratory supports, the association remained (HR 0.42; 95% CI 0.21, 0.85; *p* = 0.0160) (Fig. 1).

**Table 3:** Estimated effects of corticosteroid treatment on 60-day mortality in patients with ARDS associated with COVID-19

	Nos.	Hazard ratio	95% CI	*p *value
All ARDS patients				
Full multivariate model^a^	355	0.421	0.21, 0.85	0.0160
Sensitivity analysis				
ARDS patients defined by Berlin definition	168	0.43	0.21, 0.88	0.0208
Initiated ≤ 2 days after hospital admission versus no corticosteroids (reference)	262	0.37	0.18, 0.76	0.0072
Full multivariate model, ARDS onset as baseline^b^	335	0.48	0.24, 0.97	0.0399
Initiated ≤ 2 days after ARDS onset versus no corticosteroids, ARDS onset as baseline^b^ (reference)	279	0.45	0.22, 0.92	0.0275

All of the models assessed the effects of corticosteroids as a time-varying covariate

*ARDS* acute respiratory distress syndrome, *FIO*_*2*_ fraction of inspired oxygen, *SOFA* sequential organ failure assessment, *SpO*_*2*_ pulse oxygen saturation

^a^Adjusted for age, sex, SOFA score at hospital admission, propensity score of corticosteroid treatment, comorbidities (diabetes, hypertension, chronic pulmonary disease, chronic renal or liver disease, solid malignant tumor, hematologic malignancy, and immunosuppressive status), antiviral treatment (Lopinavir–Ritonavir, oseltamivir, and ganciclovir), and respiratory supports (high-flow oscillation oxygen, noninvasive mechanical ventilation, and invasive mechanical ventilation). Propensity score was calculated by a non-parsimonious logistic regression model that included: age, sex, SOFA score, temperature, respiratory rate, SpO_2_/FiO_2_ ratio, blood lymphocyte count, blood neutrophil count, and level of c-reactive protein at hospital admission

^b^Using values of SpO_2_/FiO_2_ ratio, respiratory rate, temperature, heart rate, respiratory rate, SOFA score, blood lymphocyte count, blood neutrophil count, and level of CRP at ARDS onset

In sensitivity analysis, narrowing to patients meet the Berlin definition of ARDS did not alter the association between corticosteroids and lower risk of death (HR 0.43; 95% CI 0.21, 0.88; *p* = 0.0208). The HR of corticosteroids on risk of death was constant when using hospital admission as baseline and excluding patients received corticosteroids 2 days after hospital admission (HR 0.37; 95% CI 0.18, 0.76; *p* = 0.0072). When using ARDS onset date as baseline, the associations between corticosteroids and the risk of death were also significant (Table 3).

Viral shedding was observed in 49.2% (188/382) of the whole population, including 69.3% (124/179) of survivors and 31.5% (64/203) of the non-survivors. In Cox regression model, we found no difference in time to viral shedding between corticosteroids-treated group and the corresponding group (HR 1.43, 95% CI 0.43, 4.80; *p* = 0.5593).

**Fig. 2:**
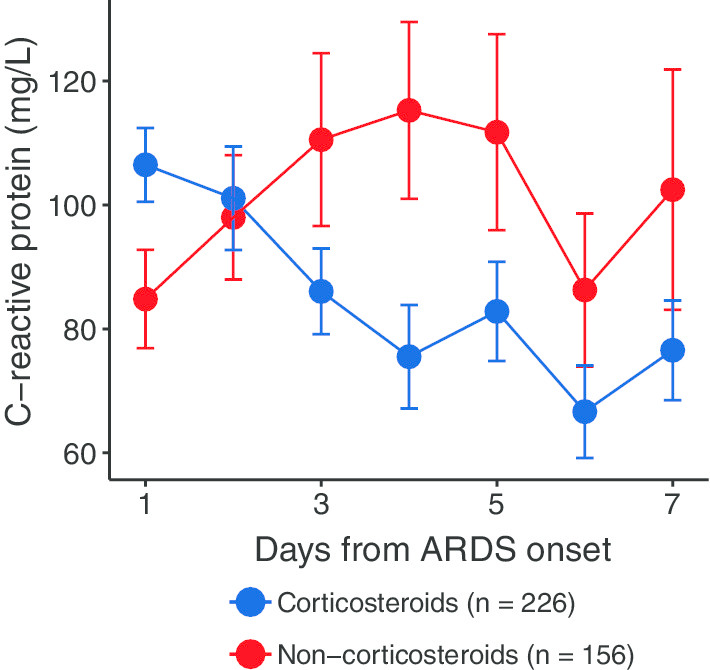
Changes in c-reactive protein in patients with ARDS associated with coronavirus disease 2019. ARDS, acute respiratory distress syndrome. Values are means ± SD

Blood CRP level decreased among corticosteroids group on the first 4 days after ARDS onset (Fig. 2), while an increase was found in non-corticosteroids group. CRP levels were significantly lower in corticosteroids-treated group after 2 days of ARDS onset (*p* for interaction = 0.0434).

## Discussion

In this observational study, prescription of low-to-moderate dose systemic corticosteroids was associated with lower risk of 60-day in-hospital death among COVID-19 patients who developed ARDS. The efficiency of corticosteroids was further supported by the reduction of CRP, as the marker for suppressed systemic inflammation responses. No associations between corticosteroid treatment with viral shedding were found in our study.

Our study demonstrates the association between corticosteroid treatment with long-term (60 days) risk of death in severe COVID-19 patients. It is biologically plausible that suppression of inflammatory response by corticosteroids may be beneficial for patients with ARDS, which was caused by dysregulated systemic inflammation [3–5] and proved the main cause of death. The RECOVERY trial and a metanalysis of ongoing RCTs showed reduced 28-day mortality and longer ventilation-free days in patients with corticosteroid treatment [13]. We used 60-day in-hospital death as primary endpoint. To our knowledge, it was longer than previously reported RCTs. The results showed significant association between corticosteroids and risk of death, which further provide evidence on the long-term benefit of corticosteroids. It was compatible with studies that indicated corticosteroid treatment was not associated with increases in secondary infections in COVID-19-related ARDS patients [15, 16].

Type, dosage, and duration of corticosteroids therapy were fundamental variables of corticosteroid treatment regimens. Different from the most of the published RCTs using dexamethasone, most of the patients in our study were treated with methylprednisolone. Methylprednisolone is a rapid onset glucocorticoid with shorter half-life and less mineralocorticoid effects than dexamethasone, which indicate shorter effects on systemic immunity and preventing corticosteroids-related fluid retention. Meduri et al. [22] firstly promoted the early use of corticosteroids in ARDS. They found that methylprednisolone reduced the duration of mechanical ventilation, ICU stay, and ICU mortality in early severe ARDS patients. In our study, a similar maximum dose (equivalent to methylprednisolone of 1–2 mg/kg) of corticosteroids was used, which was also close to the dose in RECOVERY trial and recommended by Society of Critical Care Medicine (SCCM) and European Society of Intensive Care Medicine (ESICM) for ARDS patients [23, 24]. Our results were in line with the RCTs of COVID-19 [13, 14] and previous studies of other ARDS patients, which showed low-dose corticosteroid treatment (equivalent to methylprednisolone of 1–2 mg/kg) accelerates the resolution of ARDS [23, 25, 26], indicating low-dose methylprednisolone as an alternative to dexamethasone in COVID-19-related ARDS. Higher dose may increase risks of immune-suppression and corticosteroid-induced complications [27–29]. In this cohort, tapering strategy was performed as has been suggested by the guidelines for the ARDS-related corticosteroid insufficiency (CIRCI) to reduce deterioration from the development of a reconstituted inflammatory response and febrile response. Of note, a randomized trial included mild COVID-19 patients using a similar dose and shorter course of methylprednisolone (0.5 mg/kg twice daily for 5 days) than in the regimens of our study found no benefit on mortality. More research is needed to determine the best duration of corticosteroid therapy. A recent analysis of four trials showed prolonged corticosteroids therapy reduced mortality [25]. However, chronic side effects of corticosteroids including secondary infection and osteoporosis may occur in prolonged course of treatment.

Delayed virus clearance was reported in corticosteroid-treated patients with both SARS, MERS, and influenza [30–32], which was a major concern for the immune suppressive effects of corticosteroids, albeit its uncertain clinical relevance. We found no difference in viral shedding duration from symptom onset between corticosteroid and non-corticosteroid groups, which may explain the heterogeneity in efficacy of corticosteroids between COVID-19 and other virus infections. Of note, positive SARS-CoV-2 test results have been reported after two consecutive negative results [33]. Viral tests of throat swabs were not monitored after two consecutive negative tests in our cohort. More evidence is needed for assessing the effects of corticosteroids on clearance of SARS-CoV-2 RNA.

We used rigorous statistical method to control for survival and indication bias. Survivors-treated bias exists in observational studies that assess exposure after the start of follow-up, where only patients survived long enough had an opportunity to receive the intervention. Therefore, the patients died early are more likely to be misclassified to the no-treatment group, leading to overestimation of the effectiveness of medicine [34]. This study was specifically designed to address survivors-treated bias of corticosteroid treatment, by using a time-dependent variable for corticosteroids initiation to define corticosteroids group and non-corticosteroids group [35]. In addition, there was a propensity of clinicians to give corticosteroids to patients who were critically ill in non-randomized clinical condition. The imbalance in baseline characteristics may introduce confounders in comparison of mortality between corticosteroids and non-corticosteroids group. In this cohort, lymphopenia and elevation of CRP and lactate dehydrogenase levels were more severe in patients who received corticosteroids therapy. Propensity score is a validated method to account for baseline confounding and control selection bias in this case [36]. In this study, we performed a rigorous propensity adjustment analysis accounting for the baseline variables related to propensity of corticosteroid treatment. These added to the strength of our results that found associations between corticosteroid treatment and risk of death.

Our study had some limitations. First, unlike randomized controlled trials, the selection bias and potential confounding effects might exist. We used propensity analysis rather than standard multivariable analysis to rigorously adjust for selection bias, and time-dependent model to avoid survivors-treated bias. Nonetheless, only measured factors were controlled for due to the nature of observational study design. Second, secondary infections were not monitored in this study, because microbiological culture results needed for definite diagnosis of secondary infection were possibly affected by antibiotic treatment the patients received simultaneously. To include the delayed effects of secondary infections on mortality, a longer follow-up period of 60 days was used. Third, this study was single center and patients were sicker and transferred from other hospital, so might lacking of generality. Forth, some of the patients were treated with Lopinavir–Ritonavir; it might be a confounder because the efficacy of Lopinavir–Ritonavir in COVID-19 was unclear. Fifth, our cohort was collected in the early outbreak of COVID-19; thus, the mortality was relatively higher than other studies, which limited generalization of our results.

## Conclusion

Our findings suggest administration of low dose of corticosteroids might reduce the risk of death in COVID-19 patients who developed ARDS.

## Supplementary information

**Additional file 1:** eMethods, eTable 1 and 2, and eFigure 1 were included.

## Data Availability

The corresponding authors had full access to all of the data in the study and take responsibility for the integrity of the data and the accuracy of the data analysis. The data are available from the corresponding authors upon reasonable request.

## Abbreviations

ARDS: Acute respiratory distress syndrome
CI: Confidence intervals
COVID-19: Coronavirus disease 2019
CRP: c-reactive protein
ECMO: Extracorporeal membrane oxygenation
FIO_2_: Fraction of inspired oxygen
HR: Hazard ratios
IMV: Invasive mechanical ventilation
IQR: Interquartile range
MERS: Middle East respiratory syndrome
MV: Mechanical ventilation
NIMV: Noninvasive mechanical ventilation
SARS-CoV-2: Severe acute respiratory syndrome coronavirus 2
SD: Standard deviation
SOFA: Sequential organ failure assessment
SpO_2_: Pulse oxygen saturation
