# Supplementary material for: Corticosteroid therapy for coronavirus disease 2019-related acute respiratory distress syndrome: a cohort study with propensity score analysis
Source: Crit Care. 2020 Nov 10;24:643. doi: 10.1186/s13054-020-03340-4 (PMC7655069; doi:10.1186/s13054-020-03340-4)
Supplement: Supplementary file 1 — Additional file 1: eMethods, eTable 1 and 2, and eFigure 1 were included. [file 13054_2020_3340_MOESM1_ESM.docx]

**Additional file 1**

**eMethods**

**Diagnosis of ARDS for COVID-19 disease by WHO**(1)

Onset: within 1 week of a known clinical insult (i.e. pneumonia) or new or worsening respiratory symptoms.

Chest imaging: (radiograph, CT scan, or lung ultrasound): bilateral opacities, not fully explained by volume overload, lobar or lung collapse, or nodules.

Origin of pulmonary infiltrates: respiratory failure not fully explained by cardiac failure or fluid overload. Need objective assessment (e.g. echocardiography) to exclude hydrostatic cause of infiltrates/oedema if no risk factor present.

Oxygenation impairment in adults: Mild ARDS: 200 mmHg < PaO_2_/FiO_2_ ≤ 300 mmHg (with PEEP or CPAP ≥ 5 cmH2O); Moderate ARDS: 100 mmHg < PaO_2_/FiO_2_ ≤ 200 mmHg (with PEEP ≥ 5 cmH2O); Severe ARDS: PaO_2_/FiO_2_ ≤ 100 mmHg (with PEEP ≥ 5 cmH2O).

**Definitions**

Severity of illness was assessed on the basis of Sequential Organ Failure Assessment (SOFA) score at hospital admission and on the day of ARDS diagnosis. Immunosuppression was defined as having human immunodeficiency virus infection, hematological malignant tumor, cirrhosis, chronic renal disease, organ transplantation, or under immunosuppressor treatment within 30 days before admission. Chronic pulmonary diseases included chronic obstructive pulmonary disease, asthma, chronic bronchitis, bronchiectasis, and pulmonary tuberculosis. Lymphopenia was defined as any value below 1.0 × 10^9^/L. Hyperglycemia was defined as a glucose level >200 mg/dL at any time of the day.(2) Viral shedding were defined as continuously twice negative results of SARS-CoV-2 detection from throat-swab specimens. Durations of viral shedding were calculated as days from symptom onset to first negative test.

**eTables**

**eTable 1 Characteristics of 382 patients with ARDS associated with COVID-19, stratified by outcome**

| Characteristics | All | Non-survivors | Survivors | *P-*value |
| --- | --- | --- | --- | --- |
| N(%) | **382 (100.0)** | **203 (53.1)** | **179 (46.9)** |  |
| Age, year | **60.7 ± 14.1** | **59.1 ± 14.0** | **63.0 ± 14.0** | **0.0077** |
| Male sex | **234 (61.3)** | **128 (63.1)** | **106 (59.2)** | **0.4425** |
| Smoking history | **35 (9.2)** | **18 (8.9)** | **17 (9.5)** | **0.8313** |
| Days from onset at admission | **11.0 (8.0 - 15.0)** | **10.0 (7.0 - 14.0)** | **12.0 (9.0 - 16.5)** | **0.0029** |
| Medical history |  |  |  |  |
| Chronic pulmonary diseases | 20 (5.2) | 15 (7.4) | 5 (2.8) | 0.0442 |
| Hypertension | 136 (35.6) | 89 (43.8) | 47 (26.3) | 0.0003 |
| Diabetes | 67 (17.5) | 46 (22.7) | 21 (11.7) | 0.0051 |
| Chronic liver diseases | 15 (3.9) | 9 (4.4) | 6 (3.4) | 0.5871 |
| Chronic renal diseases | 6 (1.6) | 4 (2.0) | 2 (1.1) | 0.5034 |
| Cardiovascular diseases | 28 (7.3) | 22 (10.8) | 6 (3.4) | 0.0051 |
| Malignant tumor | 12 (3.1) | 9 (4.4) | 3 (1.7) | 0.1231 |
| Hematological malignant tumor | 2 (0.6) | 2 (1.2) | 0 (0.0) | 0.1548 |
| Immunosuppressive conditions | 14 (3.7) | 11 (5.4) | 3 (1.7) | 0.0521 |
| SOFA score at admission | **2.0 (2.0 - 3.0)** | **2.0 (2.0 - 3.0)** | **2.0 (2.0 - 3.0)** | **0.1393** |
| Corticosteroid therapy before admission | **40 (10.5)** | **18 (8.9)** | **22 (12.3)** | **0.2755** |
| Temperature on admission, ℃ | **36.8 ± 0.7** | **36.9 ± 0.8** | **36.7 ± 0.5** | **0.0069** |
| Heart rate, min^-1^ | **90.9 ± 15.3** | **92.9 ± 16.3** | **88.0 ± 13.1** | **0.0018** |
| Respiratory rate, min^-1^ | **24.0 ± 6.3** | **24.5 ± 7.1** | **23.3 ± 5.0** | **0.0609** |
| Laboratory findings at admission |  |  |  |  |
| Blood leukocyte count, × 10^9^/L | 8.1 (5.2 - 11.3) | 8.4 (5.1 - 11.7) | 7.5 (5.4 - 10.0) | 0.2593 |
| Lymphocyte count, × 10^9^/L | 0.7 (0.5 - 1.0) | 0.6 (0.5 - 0.8) | 0.8 (0.6 - 1.1) | <.0001 |
| Neutrophil count, × 10^9^/L | 6.9 (4.0 - 10.2) | 7.4 (4.2 - 10.7) | 5.7 (4.0 - 8.7) | 0.0508 |
| SpO_2_/FiO_2_ | 229.3 (175.5 - 352.4) | 218.6 (170.0 - 366.7) | 241.5 (184.0 - 332.8) | 0.2133 |
| CRP, mg/L | 89.0 (38.0 - 159.9) | 96.7 (45.9 - 160.0) | 68.7 (28.6 - 138.5) | 0.0026 |
| D-dimer, mg/L | 1.5 (0.7 - 8.0) | 1.5 (0.6 - 9.5) | 1.5 (0.7 - 7.1) | 0.9913 |
| Lactate dehydrogenase, U/L | 409.0 (304.0 - 545.0) | 429.0 (320.0 - 569.0) | 386.5 (277.0 - 509.5) | 0.0124 |
| Bilateral involvement | 358 (93.7) | 190 (93.6) | 168 (93.9) | 0.9172 |
| Corticosteroid therapy | **226 (59.2)** | **135 (66.5)** | **91 (50.8)** | **0.0019** |
| Antivirus drugs |  |  |  |  |
| Lopinavir | 91 (24.0) | 46 (22.9) | 45 (25.3) | 0.5859 |
| Ganciclovir | 32 (8.4) | 10 (5.0) | 22 (12.4) | 0.0099 |
| Interferon | 103 (27.2) | 57 (28.4) | 46 (25.8) | 0.5827 |
| Oseltamivir | 64 (16.9) | 24 (11.9) | 40 (22.5) | 0.0063 |
| Respiratory support |  |  |  |  |
| High-frequency oscillation ventilation | 146 (38.8) | 104 (52.5) | 42 (23.6) | <.0001 |
| NIMV | 147 (38.5) | 134 (66.0) | 13 (7.3) | <.0001 |
| IMV | 94 (24.6) | 90 (44.3) | 4 (2.2) | <.0001 |
| ECMO | 11 (2.9) | 11 (5.4) | 0 (0.0) | 0.0016 |
| Hyperglycemia | **32 (8.4)** | **25 (12.3)** | **7 (3.9)** | **0.0031** |
| In-hospital days | **12.0 (7.0 - 18.0)** | **14.0 (9.0 - 21.0)** | **10.0 (6.0 - 13.0)** | **<.0001** |
| Duration of viral shedding from symptom onset, day | **18.0 (14.0 - 23.0)** | **18.0 (14.0 - 23.25)** | **18.0 (14.0 - 23.0)** | **0.9704** |

*Notes:* Data are n (%) or mean (SD). For continuous variables, t-test or Mann-Whitney U test was used to calculate the P value unless otherwise noted. For categorical variables, chi-square test was used to calculate the P value unless otherwise noted.ARDS was defined according to World Health Organization interim guidance.

Abbreviations: ARDS, acute respiratory distress syndrome; SOFA, sequential Organ Failure Assessment; CRP, c-reactive protein; MV, mechanical ventilation; NIMV, non-invasive mechanical ventilation; IMV, invasive mechanical ventilation; ECMO, extracorporeal membrane oxygenation; SpO2, pulse oxygen saturation; FIO2, fraction of inspired oxygen.

**eTable 2 Characteristics of 382 patients with ARDS associated with COVID-19, stratified by corticosteroid treatment and propensity score**

| **Propensity score quartiles** | **≤ Q1** | | | | **Q1 - Q2** | | | | **Q2 - Q3** | | | | **> Q3** | | | |
| --- | --- | --- | --- | --- | --- | --- | --- | --- | --- | --- | --- | --- | --- | --- | --- | --- |
| **Groups** | **All** | **Steroids** | **No steroids** | ***P*- value** | **All** | **Steroids** | **No steroids** | ***P*- value** | **All** | **Steroids** | **No steroids** | ***P*-value** | **All** | **Steroids** | **No steroids** | ***P*- value** |
| **Observed, N(%)** | 91 (100.0) | 36 (39.6) | 55 (60.4) |  | 90 (100.0) | 45 (50.0) | 45 (50.0) |  | 90 (100.0) | 53 (58.9) | 37 (41.1) |  | 90 (100.0) | 78 (86.7) | 12 (13.3) |  |
| **Age, Mean (SD)** | 66.4 ± 12.5 | 67.5 ± 10.7 | 65.7 ± 13.6 | 0.502 | 64.1 ± 14.6 | 63.7 ± 14.9 | 64.5 ± 14.5 | 0.8134 | 59.2 ± 12.4 | 59.3 ± 11.6 | 59.2 ± 13.5 | 0.9695 | 53.8 ± 13.6 | 53.1 ± 13.8 | 58.8 ± 11.8 | 0.1795 |
| **Male sex, (%)** | 36 (39.6) | 15 (41.7) | 21 (38.2) | 0.7396 | 45 (50.0) | 23 (51.1) | 22 (48.9) | 0.833 | 62 (68.9) | 38 (71.7) | 24 (64.9) | 0.4908 | 76 (84.4) | 64 (82.1) | 12 (100.0) | 0.1103 |
| **SOFA score at admission, Median (IQR)** | 2.0 (0.0 - 2.0) | 2.0 (0.0 - 2.5) | 2.0 (2.0 - 2.0) | 0.9753 | 2.0 (2.0 - 3.0) | 2.0 (2.0 - 3.0) | 2.0 (2.0 - 2.0) | 0.104 | 2.0 (2.0 - 3.0) | 2.0 (2.0 - 3.0) | 2.0 (2.0 - 3.0) | 0.6397 | 2.0 (2.0 - 4.0) | 2.0 (2.0 - 4.0) | 2.5 (2.0 - 6.0) | 0.4407 |
| **Temperature on admission, ℃** | 36.5 ± 0.4 | 36.5 ± 0.4 | 36.6 ± 0.4 | 0.2783 | 36.6 ± 0.5 | 36.6 ± 0.5 | 36.7 ± 0.5 | 0.6274 | 36.8 ± 0.6 | 36.8 ± 0.6 | 36.8 ± 0.6 | 0.9902 | 37.3 ± 1.0 | 37.4 ± 1.0 | 37.1 ± 0.8 | 0.3708 |
| **Heart rate, min^-1^** | 82.6 ± 11.6 | 81.9 ± 14.3 | 83.1 ± 9.5 | 0.6175 | 87.0 ± 13.4 | 88.4 ± 15.2 | 85.6 ± 11.4 | 0.3214 | 89.8 ± 11.9 | 89.2 ± 12.1 | 90.8 ± 11.8 | 0.5259 | 102.5 ± 15.6 | 102.7 ± 16.1 | 100.9 ± 12.1 | 0.7075 |
| **Respiratory rate, min^-1^** | 21.6 ± 3.7 | 21.6 ± 4.6 | 21.6 ± 3.1 | 0.9795 | 23.2 ± 4.5 | 23.4 ± 4.2 | 23.1 ± 4.8 | 0.8151 | 24.5 ± 5.6 | 24.1 ± 5.5 | 25.1 ± 5.8 | 0.393 | 26.6 ± 9.3 | 27.0 ± 9.7 | 24.0 ± 5.4 | 0.2982 |
| **Lymphocyte count, × 10^9^/L** | 1.0 (0.7 - 1.5) | 0.9 (0.7 - 1.2) | 1.1 (0.8 - 1.6) | 0.0408 | 0.7 (0.5 - 1.0) | 0.6 (0.5 - 0.8) | 0.7 (0.5 - 1.0) | 0.8181 | 0.6 (0.5 - 0.8) | 0.6 (0.5 - 0.7) | 0.6 (0.5 - 0.9) | 0.0992 | 0.5 (0.4 - 0.7) | 0.5 (0.4 - 0.7) | 0.5 (0.4 - 0.7) | 0.6691 |
| **Neutrophil count, × 10^9^/L** | 4.9 (3.4 - 7.8) | 5.8 (3.7 - 10.8) | 4.7 (3.3 - 6.6) | 0.042 | 7.4 (4.1 - 9.6) | 7.5 (3.5 - 9.6) | 7.4 (4.8 - 9.5) | 0.6168 | 7.0 (4.6 - 9.7) | 7.3 (4.0 - 8.7) | 6.9 (5.4 - 10.6) | 0.4481 | 8.2 (4.5 - 13.6) | 8.3 (4.5 - 13.5) | 7.9 (3.7 - 16.1) | 0.9338 |
| **SpO_2_/FiO_2_** | 264.9 (190.0 - 414.3) | 237.1 (166.1 - 426.2) | 275.8 (213.3 - 338.1) | 0.7362 | 225.6 (178.0 - 317.2) | 224.4 (160.0 - 317.2) | 229.7 (182.0 - 296.6) | 0.8149 | 221.1 (170.0 - 381.0) | 206.7 (170.0 - 376.2) | 236.6 (180.0 - 381.0) | 0.7741 | 200.0 (170.0 - 371.4) | 200.0 (170.0 - 371.4) | 242.0 (165.0 - 397.5) | 0.7128 |
| **CRP, mg/L** | 40.7 (12.1 - 80.1) | 49.9 (28.0 - 92.3) | 31.3 (5.7 - 66.6) | 0.0138 | 80.0 (40.0 - 155.0) | 73.0 (40.3 - 125.1) | 107.9 (40.0 - 160.0) | 0.3182 | 114.2 (58.7 - 160.0) | 109.8 (55.3 - 159.9) | 123.3 (80.5 - 160.0) | 0.5891 | 134.7 (82.0 - 160.0) | 143.4 (83.2 - 160.0) | 95.4 (67.3 - 158.6) | 0.3048 |

*Note:* Propensity score was calculated by a non-parsimonious logistic regression model that included: age; sex; SOFA score at admission; temperature, respiratory rate, SpO_2_/FiO_2_ ratio, blood lymphocyte count, blood neutrophil count, and level of c-reactive protein at admission.

Abbreviations: SOFA, sequential Organ Failure Assessment; SpO_2_, pulse oxygen saturation; FiO_2_, fraction of inspired oxygen

**eFigures**

**eFigure 1 Workflow of the Selection process for patients with ARDS associated with COVID-19**

**
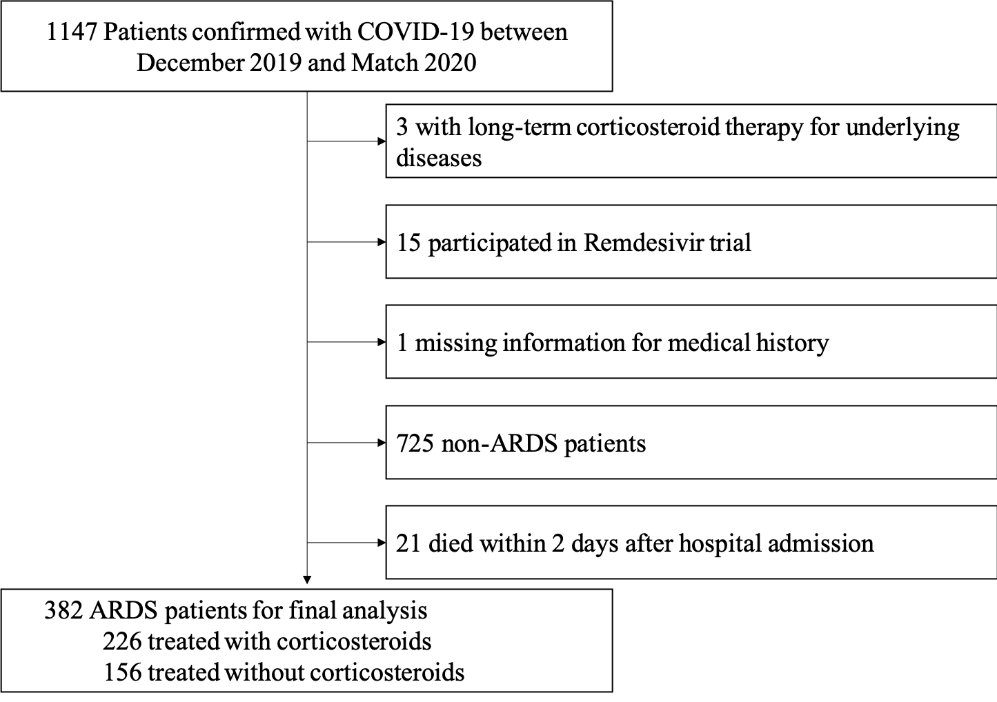
**

Abbreviations: COVID-19= coronavirus disease 2019, ARDS=acute respiratory distress syndrome. Values were mean ± SD.

**Reference**

1. Organization WH. Clinical management of COVID-19, interim guidance, 27 May 2020. [cited 2020 March 13, 2020]Available from:

2. Perez A, Jansen-Chaparro S, Saigi I, et al. Glucocorticoid-induced hyperglycemia. J Diabetes 2014;6(1):9-20.
